# A comparative study of wavelet families for schizophrenia detection

**DOI:** 10.3389/fnhum.2024.1463819

**Published:** 2024-12-10

**Authors:** E. Sathiya, T. D. Rao, T. Sunil Kumar

**Affiliations:** ^1^Department of Mathematics, School of Advanced Sciences, Vellore Institute of Technology, Chennai, India; ^2^Department of Electrical Engineering, Mathematics and Science, University of Gävle, Gävle, Sweden

**Keywords:** schizophrenia, discrete wavelet transform, EEG classification, statistical features, decomposition level

## Abstract

Schizophrenia (SZ) is a chronic mental disorder, affecting approximately 1% of the global population, it is believed to result from various environmental factors, with psychological factors potentially influencing its onset and progression. Discrete wavelet transform (DWT)-based approaches are effective in SZ detection. In this report, we aim to investigate the effect of wavelet and decomposition levels in SZ detection. In our study, we analyzed the early detection of SZ using DWT across various decomposition levels, ranging from 1 to 5, with different mother wavelets. The electroencephalogram (EEG) signals are processed using DWT, which decomposes them into multiple frequency bands, yielding approximation and detail coefficients at each level. Statistical features are then extracted from these coefficients. The computed feature vector is then fed into a classifier to distinguish between SZ and healthy controls (HC). Our approach achieves the highest classification accuracy of 100% on a publicly available dataset, outperforming existing state-of-the-art methods.

## 1 Introduction

Schizophrenia (SZ) is characterized by various symptoms such as hallucinations, delusions, altered perception, negative emotional states, and cognitive deficits often resulting in fearfulness, withdrawal, and social difficulty (Guo et al., 2021). Although the exact cause of SZ is still unknown, it is thought to be caused by environmental factors that increase the risk of SZ, particularly during the prenatal or perinatal periods, long before the typical onset of symptoms in late adolescence or early adulthood (Guo et al., 2021). In order to diagnose individuals with SZ, clinical psychiatrists use clinical interviews, screening, and testing methods, also it is important for a psychiatrist to conduct a comprehensive examination to exclude substance misuse or other neurological disorders with symptoms that resemble SZ (Ranjan et al., 2024). However, these conventional methods provide subjective results, are time-consuming, and are sensitive to errors. Therefore, early and timely intervention for SZ could help reduce the disease progression by addressing it in its earlier stage (Ranjan et al., 2024).

In recent years, electroencephalogram (EEG) has emerged as a powerful diagnostic tool for brain disorders due to its non-invasiveness, objectivity, low cost, minimal time requirement, and lack of radiation exposure (Li et al., 2023; Sharma and Joshi, 2021; Zhou et al., 2018; Safi and Safi, 2021; Wijaya et al., 2015). Various machine learning (ML) techniques have been extensively used for SZ and healthy control (HC) classification with EEG signals. These techniques effectively handle the non-stationary nature of EEG data by extracting critical time-frequency patterns. For instance, the authors in Khare et al. (2020) decomposed EEG signals into modes using empirical wavelet transformation (EWT) and extracted linear and non-linear time-domain features from these modes. They concluded that the first two amplitude mode (AM)-frequency mode (FM) components provide the most information for diagnosing SZ and HC.

In Khare and Bajaj (2021), the authors developed flexible-tunable Q wavelet transform (F-TQWT) for efficient feature extraction, which is then fed into the flexible least square support vector machine (F-LSSVM) classifier for automatic tuning of hyper-parameters, aimed at improving the discrimination between SZ and HC. In Gosala et al. (2023), the authors conducted a comparative study using wavelet scattering transform (WST), continuous wavelet transform (CWT), and discrete wavelet transform (DWT) for the classification of SZ and HC. In Agarwal and Singhal (2023), the authors used a fast Fourier transform (FFT) to divide a signal into sub-band (SB) components, from which statistical features were computed. Additionally, they developed a look-ahead pattern (LAP) feature to capture local variations in the EEG signal for SZ detection.

In Ruiz de Miras et al. (2023), the authors extracted both linear and non-linear features, which were then combined using principal component analysis (PCA) and provided as input to different classifiers for SZ detection. The observed differences were primarily localized in a specific region of the right brain hemisphere, particularly the opercular area and temporal lobe. In Kumar et al. (2023), the authors utilized a histogram of local variance (HLV) and symmetrically weighted local binary pattern (SLBP)-based automated approach for detecting SZ in adolescents from EEG signals to discriminate between SZ and HC. In Siuly et al. (2020), the authors decomposed EEG signals into intrinsic mode functions (IMFs) from empirical mode decomposition (EMD) and extracted statistical features for the detection of SZ. In Krishnan et al. (2020), the authors introduced a multivariate (EMD)-based approach in which the randomness of the IMF signal was assessed by computing its entropy measures, demonstrating a significant distinction between HC and SZ. In Baygin et al. (2023), the authors utilized a carbon chain pattern (CCP) model with iterative tunable Q-factor wavelet transform (ITQWT), and clinically significant features were selected using iterative neighborhood component analysis (INCA) for SZ detection. In Baygin et al. (2021), the authors proposed a novel Collatz conjecture-based model for the classification of SZ and HC. The model utilizes maximum absolute pooling for decomposition and aims to demonstrate the feature generation capability of the conjecture-based structure. In Akbari et al. (2021), the authors developed a novel framework for diagnosing SZ using phase space dynamic (PSD) analysis and extracted 15 graphical features from PSD. Using the forward selection algorithm, they identified the optimal features and channels, with the Cz channel showing more regularity in SZ. In Das and Pachori (2021), the authors proposed univariate iterative filtering (IF) in which the EEG data are decomposed into multi-IMFs, and the Hjorth parameter is extracted as a feature for the classification of SZ and HC. In Hossein Najafzadeh et al. (2021), the authors proposed a new method based on the adaptive neuro-fuzzy inference system (ANFIS), utilizing four features: Shannon entropy (ShEn), spectral entropy (SpEn), approximate entropy (ApEn), and the absolute value of the highest slope of autoregressive coefficients (AVLSACs). This study led to the development of a new decision support system (DSS) that can receive a person’s EEG signal and distinguish between SZ patients and HCs.

More recently, deep learning (DL) techniques have gained importance in EEG-based SZ detection. These methods can automatically extract meaningful features and patterns from complex EEG data, improving diagnostic accuracy (Acc). The authors in Göker (2023) used the 1D-CNN-based channel selection mechanism, focusing on different brain regions for SZ and HC classification. In Khare et al. (2023), the authors developed a unique DL model named SchizoNET combining the Margenau-Hill time-frequency distribution with CNN for automatic SZ detection. Despite the advances of DL techniques in SZ detection, traditional methods such as wavelet transforms (WTs) still play a crucial role in their capability to capture the time-frequency characteristics of EEG signals (Gosala et al., 2023). WTs enable time-variant decomposition, allowing for different filtering settings across different time ranges and providing event-related filter responses while eliminating edge effects associated with traditional band-pass filters (Zhang et al., 2016). Over the past decade, researchers have applied various wavelet filters to represent time series data, with DWT emerging as a popular tool for EEG analysis due to its capability to capture both time and frequency domain features (Zhang et al., 2016). However, the application of DWT with different decomposition levels and wavelet types for the classification of SZ and HC remains largely unexplored. This motivated our study, where we conducted a performance analysis of DWT-based statistical features, considering multiple decomposition levels and wavelet filters for distinguishing SZ from HC.

The contributions of our analysis are as follows:

1. Utilized DWT-based statistical features, with a decomposition level up to 5, by using a combination of different mother wavelets to classify EEG signals into SZ and HC groups.
2. Validated our results using both 10-fold cross-validation and an 80:20 train-test split.
3. Our analysis demonstrated significantly improved classification performance over existing methods.

The structure of this paper is as follows: Section 2 outlines the experimental procedure used for SZ detection and presents the results and discussion. Finally, in Section 4, the conclusions are provided.

## 2 Proposed analysis

**Figure 1:**
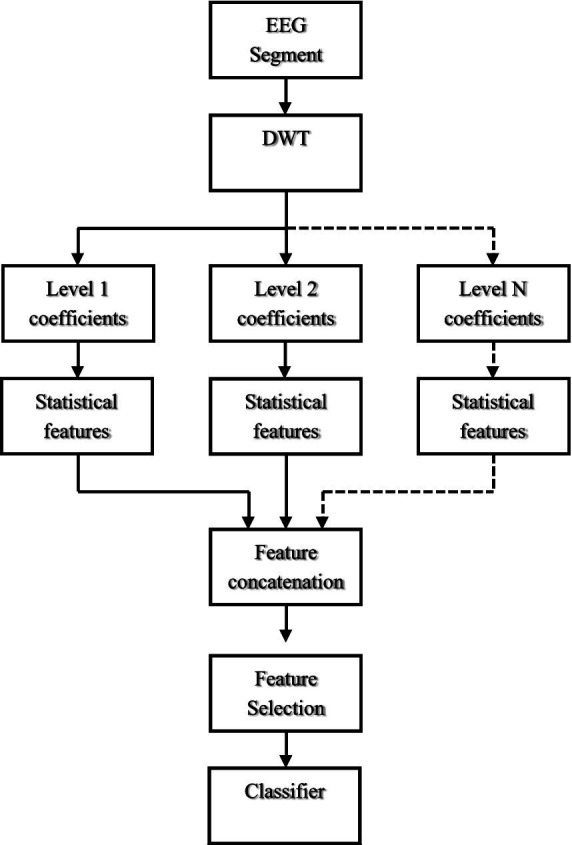
Block diagram of our approach.

In this section, Figure 1 illustrates the flowchart of our experimental analysis. Initially, the EEG signals undergo processing through DWT by decomposing them into a series of detailed and approximation coefficients at multiple scales. This transforms the non-stationary EEG signals into a multi-scale SB format. Following this, we extract statistical features from each level of wavelet coefficients. These extracted features are then concatenated and given as input to classifiers for the classification of SZ and HC. A detailed explanation of each step is provided below.

### 2.1 Discrete wavelet transform

**Figure 2:**
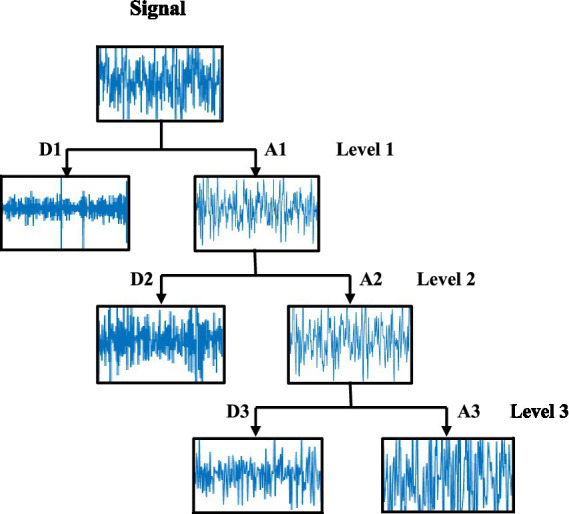
Sub-band decomposition of an EEG signal using DWT.

The discrete wavelet transform (DWT) allows the analysis of signal features in both time and frequency domains by decomposing the signal into low-frequency and high-frequency components. Each stage of this method involves a high-pass filter and a low-pass filter, with the output signals of these filters being down-sampled by a factor of 2 (Mallat, 1989; Majid Aljalal et al., 2022; Kandala et al., 2021). In the first decomposition level, a high-pass filter with down-sampling produces the detail coefficients $\left(D1\right)$, while a low-pass filter with down-sampling provides the approximation coefficients $\left(A1\right)$, and the approximation coefficients are further processed to obtain detailed and approximation coefficients of the next level. This process will be repeated again until obtaining the approximation $\left(A_{n}\right)$ and detail coefficients $\left(D_{n}\right)$ in the last level. The three-level SB decomposition of an EEG signal is shown in Figure 2.

### 2.2 Statistical features

The effectiveness of the feature extraction significantly impacts the classification performance. In our study, we extracted statistical features (Sunil Kumar and Kanhangad, 2017; Kumar et al., 2019) such as entropy (Krishnan et al., 2020), standard deviation (Siuly et al., 2020), skewness (Ruiz de Miras et al., 2023), and kurtosis (Ruiz de Miras et al., 2023) from the SB of each of the wavelet coefficients resulting from the DWT of the signal.

### 2.3 Feature concatenation and classification

In this, the feature matrix is arranged through the concatenation of the extracted statistical features from all segments. In our analysis, we utilized an ensemble classifier (Han et al., 2019).

### 2.4 Feature selection

Feature selection (FS) helps in enhancing the model’s performance better by keeping only the essential features. Chi-square calculates whether the observed values deviate significantly from the expected values if the features were independent (Theresia Diah et al., 2019), and it is given by.


(1) $$\chi 2=\sum \frac{{\left(O-E\right)}^{2}}{E}$$

where, O represents the observed frequency and E is the expected frequency.

The chi-square statistic is computed and compared to a set significance level (generally, 0.05) as described by Equation 1. If it is below this threshold, the feature is considered statistically significant and is selected for classification.

## 3 Experimental outcome

This section provides a detailed description of the dataset, followed by the results and discussion sections.

### 3.1 Dataset

This study uses an EEG dataset, publicly available on the Moscow State University website (Gorbachevskaya and Borisov, 2002), comprising 84 adolescents, including 45 individuals with SZ disorder and 39 HCs. The EEG signals were recorded from subjects aged between 10 years and 8 months and 14 years, using a 16-electrode system based on the international 10–20 placement system. The signals are sampled at a rate of 128 Hz, by experts at the Mental Health Research Center (MHRC), where none of the patients received chemotherapy during the examination. More details about this dataset can be found in Borisov et al. (2005).

### 3.2 Results

**Figure 3:**
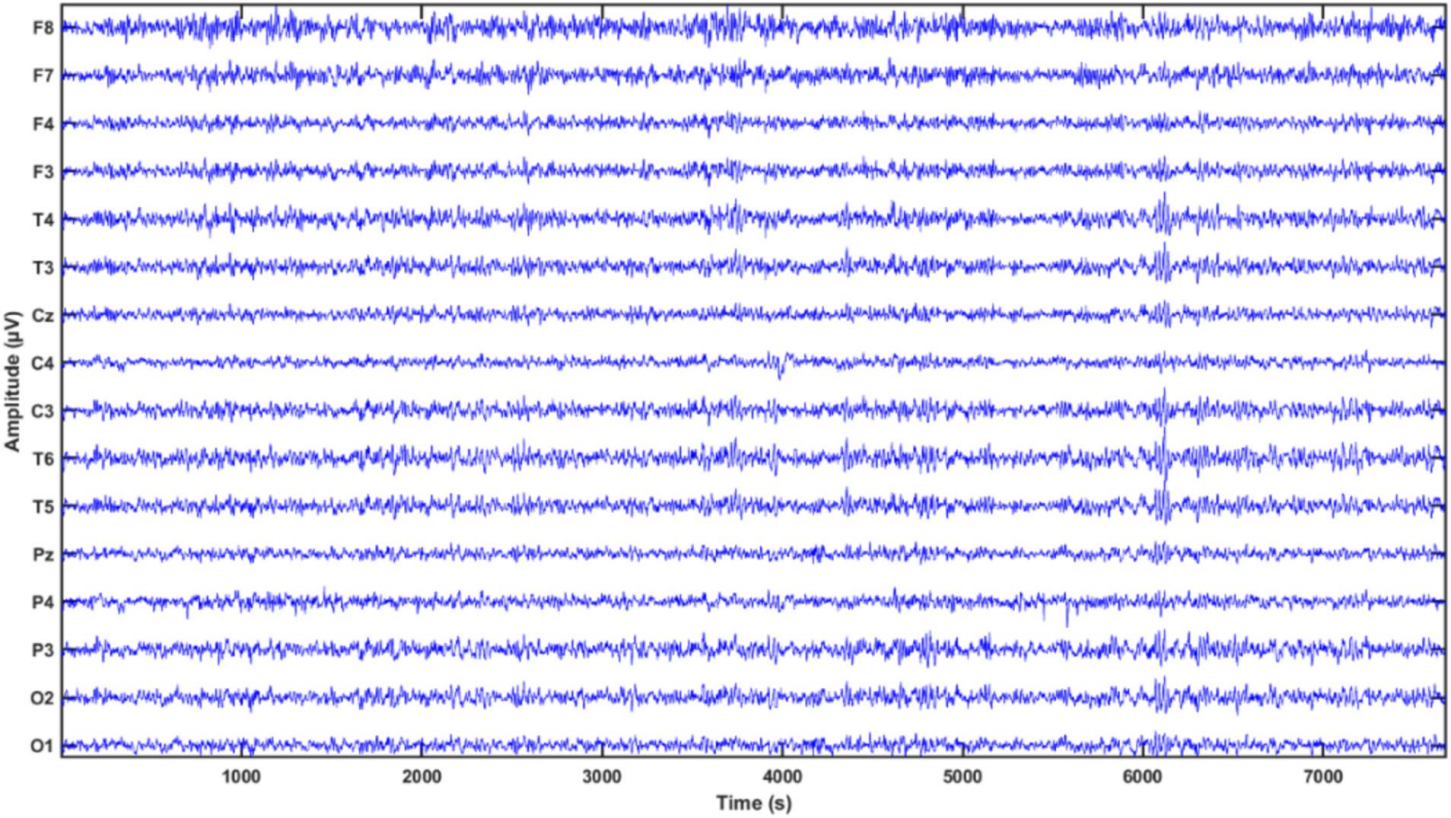
Sample plot of raw EEG signal.

Selecting the appropriate number of decomposition levels is crucial for DWT in EEG signal analysis (Subasi, 2007). The decision to use a five-level decomposition was based on the fact that EEG signals typically do not contain significant information above 30 Hz (Subasi, 2007; Sharmila and Mahalakshmi, 2017). Depending on the type of signal to be analyzed, the mother wavelet is chosen according to the convenience and the requirement of the experimenter (Gandhi et al., 2011). In our study, we used Daubechies (db) from 2 to 5 (Daubechies, 1992), Fejer-Korovkin (fk) at 4, 6, and 8 (Nielsen, 2001), biorthogonal (bior) such as 1.1, 1.3, and 1.5 (Daubechies, 1992), coiflets (coif) from 1 to 5 (Daubechies, 1992) and symlets (sym) from 2 to 5 (Daubechies, 1992). As sharp transitions are critical for capturing transient EEG patterns (Rafiee et al., 2011; Venkata Phanikrishna et al., 2021; Cheng et al., 2019) db and sym are used, coif has been used due to their time-frequency localization, its ability to detect subtle oscillatory patterns, and its large filter length (Rafiee et al., 2011; Venkata Phanikrishna et al., 2021; Cheng et al., 2019). Fejer-Korovkin (fk) is known for its enhanced symmetry but is less smooth compared to db (Nielsen, 2001). We also selected biorthogonal (bior) because it can preserve both amplitude and phase (Rafiee et al., 2011; Venkata Phanikrishna et al., 2021; Cheng et al., 2019) (see Figure 3).

To demonstrate the significance of our analysis, we conducted two experiments: a 10-fold cross-validation technique and an 80:20 ratio for training and testing. Tables 1–8 show the classification Acc across various wavelet types and decomposition levels. Tables 1–4 show accuracy for 30-s EEG segments, while Tables 5–8 show results for 60-s segments.

**Table 1:** Classification accuracy achieved with 30-s EEG segment using 10-fold cross-validation.

Classifier: Ensemble						
Validation	Wavelets	Level 1 decomposition (%)	Level 2 decomposition (%)	Level 3 decomposition (%)	Level 4 decomposition (%)	Level 5 decomposition (%)
10-fold	db2	85.7	87.5	94.6	88.7	92.9
	db3	85.7	88.7	90.5	91.7	93.5
	db4	86.3	86.3	90.5	89.9	91.7
	db5	88.1	86.3	87.5	91.1	92.9
	bior1.1	83.9	86.3	88.1	91.7	85.7
	bior1.3	83.3	88.7	88.1	88.7	88.1
	bior1.5	85.1	85.7	89.9	90.5	92.3
	fk4	85.1	86.9	89.3	91.7	92.3
	fk6	83.3	85.1	90.5	87.5	91.7
	fk8	86.9	81	88.1	92.3	89.9
	coif1	84.5	88.7	89.3	88.7	90.5
	coif2	83.9	84.5	89.3	89.3	91.1
	coif3	87.5	83.3	91.1	92.3	88.7
	coif4	86.3	83.9	88.1	90.5	91.7
	coif5	85.1	82.7	89.9	91.7	88.1
	sym2	83.9	86.9	91.1	87.5	92.9
	sym3	88.1	83	87.5	90.5	89.9
	sym4	86.3	84.5	86.3	90.5	93.5
	sym5	82.7	81	88.7	91.7	91.7

**Table 2:** Classification accuracy achieved with 30-s EEG segment using 10-fold cross-validation with chi-square FS.

Classifier: Ensemble						
Validation	Wavelets	Level 1 decomposition (%)	Level 2 decomposition (%)	Level 3 decomposition (%)	Level 4 decomposition (%)	Level 5 decomposition (%)
10-fold	db2	83.9	87.5	88.7	90.5	94
	db3	86.9	88.7	89.9	93.5	93.5
	db4	85.1	88.1	89.9	90.5	91.1
	db5	87.5	90.9	89.9	91.7	92.3
	bior1.1	83.9	83.9	88.1	90.5	86.9
	bior1.3	85.7	86.3	88.1	89.9	86.9
	bior1.5	84.5	86.3	85.7	89.3	89.9
	fk4	85.1	85.7	85.1	92.3	89.9
	fk6	83.3	87.9	88.1	90.5	94
	fk8	86.9	83.3	85.7	92.3	88.1
	coif1	86.9	89.3	89.3	92.9	92.9
	coif2	88.7	84.5	87.5	91.1	90.5
	coif3	85.1	84.5	91.1	90.5	92.9
	coif4	83.9	82.7	89.3	91.1	92.3
	coif5	83.9	83.9	88.7	92.3	89.3
	sym2	85.7	88.1	91.7	90.5	92.9
	sym3	86.3	82.7	92.9	91.1	89.9
	sym4	82.1	82.7	88.1	90.5	90.5
	sym5	86.3	81.5	91.7	91.7	94

**Table 3:** Classification accuracy achieved with 30-s EEG segment using an 80:20 split.

Classifier: Ensemble						
Validation	Wavelets	Level 1 decomposition (%)	Level 2 decomposition (%)	Level 3 decomposition (%)	Level 4 decomposition (%)	Level 5 decomposition (%)
80:20	db2	87.9	90.9	93.9	87.9	93.9
	db3	84.8	93.9	93.9	90.9	93.9
	db4	87.9	87.9	93.9	93.9	93.9
	db5	90.9	85.7	97	90.9	90.9
	bior1.1	81.8	81.8	90.9	90.9	90.9
	bior1.3	81.8	87.9	87.9	90.9	87.9
	bior1.5	81.8	90.9	97	97	90.9
	fk4	81.8	81.8	97	93.9	93.9
	fk6	90.9	87.9	90.9	90.9	90.9
	fk8	87.9	90.9	90.9	97	90.9
	**coif1**	84.8	87.9	90.9	90.9	**100**
	**coif2**	87.9	87.9	93.9	**100**	87.9
	**coif3**	93.9	84.8	91.1	93.9	**100**
	**coif4**	87.9	84.8	89.3	90.9	93.9
	coif5	87.4	87.9	88.7	93.9	**100**
	sym2	87.9	90.9	90.9	93.9	97
	sym3	87.9	90.9	87.9	93.9	90.9
	sym4	90.9	90.9	90.9	90.9	93.9
	sym5	87.9	81.8	93.9	90.9	93.9

Bold values in the tables indicate the highest performance metrics achieved for the experiment.

**Table 4:** Classification accuracy achieved with 30-s EEG segment using an 80:20 split with chi-square FS.

Classifier: Ensemble						
Validation	Wavelets	Level 1 decomposition (%)	Level 2 decomposition (%)	Level 3 decomposition (%)	Level 4 decomposition (%)	Level 5 decomposition (%)
80:20	db2	90.9	87.9	93.9	88.9	93.9
	db3	84.8	90.9	90.9	90.9	93.9
	db4	87.9	87.9	90.9	**100**	90.9
	db5	90.9	93.9	97	90.9	90.9
	bior1.1	90.9	81.8	87.9	97	87.9
	bior1.3	90.9	90.9	87.9	97	87.9
	bior1.5	87.9	93.9	87.9	93.9	97
	fk4	87.9	84.8	87.9	90.9	87.9
	fk6	87.9	87.9	93.9	93.9	90.9
	fk8	90.9	90.9	90.9	97	90.9
	coif1	87.9	90.9	90.9	97	90.9
	coif2	87.9	87.9	90.9	90.9	93.9
	coif3	87.9	84.8	90.9	97	97
	coif4	84.8	87.9	87.9	93.9	97
	coif5	87.9	90.9	97	97	90.9
	sym2	90.9	84.8	97	90.9	97
	sym3	87.9	84.8	93.9	93.9	93.9
	**sym4**	93.9	84.8	90.9	90.9	**100**
	sym5	87.9	81.8	97	93.9	97

Bold values in the tables indicate the highest performance metrics achieved for the experiment.

**Table 5:** Classification accuracy achieved with a 60-s EEG segment using 10-fold cross-validation.

Classifier: Ensemble						
Validation	Wavelets	Level 1 decomposition (%)	Level 2 decomposition (%)	Level 3 decomposition (%)	Level 4 decomposition (%)	Level 5 decomposition (%)
10-fold	db2	63.1	65.5	71.4	64.3	67.9
	db3	57.1	65.5	63.1	63.1	65.5
	db4	58.3	65.5	63.1	66.7	69
	db5	58.3	63.1	67.9	65.5	67.9
	bior1.1	59.5	64.3	65.5	61.9	61.9
	bior1.3	61.9	63.1	64.3	71.4	69
	bior1.5	60.7	63.1	65.5	65.5	71.4
	fk4	56	63.1	66.7	65.5	61.9
	fk6	58.3	71.4	63.1	66.7	61.9
	fk8	58.3	64.3	61.9	76.2	63.1
	coif1	58.3	67.9	61.9	65.5	66.7
	coif2	56	70.2	63.1	72.6	64.3
	coif3	57.1	66.7	65.5	70.2	65.5
	coif4	58.3	64.3	61.9	70.2	70.2
	coif5	59.5	60.7	61.9	71.4	69
	sym2	58.3	63.1	63.1	70.2	64.3
	sym3	58.3	70.2	65.5	64.3	61.9
	sym4	60.7	70.2	64.3	66.7	70.2
	sym5	61.9	66.7	64.3	69	70.2

**Table 6:** Classification accuracy achieved with a 60-s EEG segment using 10-fold cross-validation with chi-square FS.

Classifier: Ensemble						
Validation	Wavelets	Level 1 decomposition (%)	Level 2 decomposition (%)	Level 3 decomposition (%)	Level 4 decomposition (%)	Level 5 decomposition (%)
10-fold	db2	57.1	67.9	67.9	63.1	69
	db3	60.7	70.2	65.5	64.3	65.5
	db4	61.9	66.2	65.5	66.7	70.2
	db5	64.3	67.9	64.3	71.4	67.9
	bior1.1	64.3	61.9	64.3	61.9	63.1
	bior1.3	60.7	66.7	63.1	65.5	63.1
	bior1.5	60.7	65.5	64.3	70.2	67.9
	fk4	61.9	64.3	67.9	64.3	64.3
	fk6	63.1	69	67.9	71.4	70.2
	fk8	58.3	65.5	63.1	71.4	70.2
	coif1	60.7	64.3	65.5	71.4	70.2
	coif2	61.9	67.9	66.7	73.8	70.2
	coif3	61.9	61.9	64.3	72.6	64.3
	coif4	61.9	61.9	63.1	72.6	70.2
	coif5	63.2	63.1	61.9	72.6	70.2
	sym2	63.1	64.3	63.9	63.1	70.2
	sym3	60.7	64.3	63.1	70.2	65.5
	sym4	63.1	69	64.3	65.5	71.4
	sym5	58.3	63.1	63.1	72.6	73.8

**Table 7:** Classification accuracy achieved with a 60-s EEG segment using an 80:20 split.

Classifier: Ensemble						
Validation	Wavelets	Level 1 decomposition (%)	Level 2 decomposition (%)	Level 3 decomposition (%)	Level 4 decomposition (%)	Level 5 decomposition (%)
80:20	db2	68.8	62.5	87.5	75	75
	db3	62.5	75	62.5	81.2	68.8
	db4	62.5	75	62.5	75	68.8
	db5	62.5	68.8	75	68.8	68.8
	bior1.1	68.8	62.5	75	62.5	68.8
	bior1.3	62.5	62.5	68.8	68.8	62.5
	bior1.5	75	68.8	75	75	62.5
	fk4	62.5	75	62.5	75	68.8
	fk6	68.8	81.2	62.5	81.2	81.2
	fk8	68.8	75	75	87.5	75
	coif1	62.5	87.5	68.8	81.2	68.8
	coif2	62.5	75	62.5	75	75
	coif3	81.2	68.8	68.8	68.8	68.8
	**coif4**	75	62.5	75	**93.8**	62.5
	coif5	68.8	81.2	62.5	87.5	81.5
	sym2	68.8	81.2	62.5	62.5	81.2
	sym3	68.8	68.8	62.5	62.5	68.8
	sym4	62.5	75	62.5	62.5	75
	sym5	68.8	68.8	68.8	62.5	68.8

Bold values in the tables indicate the highest performance metrics achieved for the experiment.

**Table 8:** Classification accuracy achieved with a 60-s EEG segment using an 80:20 split with chi-square FS.

Classifier: Ensemble						
Validation	Wavelets	Level 1 decomposition (%)	Level 2 decomposition (%)	Level 3 decomposition (%)	Level 4 decomposition (%)	Level 5 decomposition (%)
80:20	db2	62.5	81.2	75	81.2	68.8
	db3	81.2	75	62.5	75	75
	db4	62.5	81.2	81.2	75	68.8
	db5	68.8	68.8	75	62.5	81.2
	bior1.1	75	81.2	62.5	81.2	62.5
	bior1.3	75	75	75	75	62.5
	bior1.5	81.2	62.5	68.8	75	68.8
	fk4	62.5	81.2	68.8	75	75
	fk6	75	75	62.5	75	75
	fk8	62.5	68.8	75	87.5	75
	coif1	62.5	75	81.2	81.2	75
	coif2	62.5	75	81.2	75	75
	coif3	62.5	68.8	75	75	68.8
	coif4	68.8	68.8	75	81.2	87.5
	coif5	68.8	68.8	75	75	75
	sym2	87.5	75	75	81.2	87.5
	**sym3**	68.8	87.5	68.8	**93.8**	68.8
	sym4	62.5	75	62.5	75	81.2
	sym5	81.2	62.5	62.5	75	75

Bold values in the tables indicate the highest performance metrics achieved for the experiment.

In our study, we evaluated various wavelet types and decomposition levels for SZ and HC classification using EEG signals across 30 and 60-s segments. For 30-s segments, coif wavelets performed best without FS, achieving 100% Acc at levels 4 and 5 in the 80:20 split method (Table 3). With FS, sym4 and db4 wavelets reached 100% Acc at level 4 (Table 4). For 60-s segments, coif4 achieved 93.8% Acc at level 4 in both 10-fold cross-validation and the 80:20 split method (Tables 7, 8). FS further optimized the classification, especially for sym4 and db4, maintaining an Acc of 93.8%.

Overall, the utilization of chi-square FS improved the feature vector selection, which led to enhanced classification Acc, particularly with the sym and coif wavelets. These wavelets were particularly effective for SZ detection. Furthermore, level 4 decomposition proved most effective for detecting SZ in both 30 and 60-s data segments.

### 3.3 Discussion

Our proposed approach achieved 100% Acc, outperforming several existing methods, as shown in Table 9, by using statistical features extracted from the DWT of the EEG signal. DWT’s ability to capture both time and frequency characteristics of non-stationary EEG signals makes it effective for SZ detection. By carefully, exploring different wavelet families and decomposition levels, we demonstrated that the multi-resolution analysis provided by DWT offers a more robust classification performance. The authors in Kumar et al. (2023), Gosala et al. (2023), and Khare and Bajaj (2022), have used the same dataset as ours, while others have utilized different datasets and various feature extraction methods for the classification of SZ and HC. The authors in Khare and Bajaj (2021) extracted time-frequency features using F-TQWT. The authors in Krishnan et al. (2020), Siuly et al. (2020), and Das and Pachori (2021) have used EMD of IMF-7-based statistical features, MEMD for extracting the entropy measures, and proposed MIF, an extension of univariate IF for multivariate signals, to decompose multi-channel EEG data into IMFs, from which features such as Hjorth parameters are computed in detecting SZ. In general, EMD has computational complexity. Our study, using DWT with multiple wavelet families, is a more computationally efficient method. The authors in Gosala et al. (2023) have used various wavelet methods such as CWT, DWT, and WST and extracted time-domain, frequency-domain, and time-frequency domain features from these wavelet coefficients. The authors in Sharma and Acharya (2021) implemented seven wavelet-based $l_{1}$ norm features using single-channel Cz and orthogonal wavelet filters. In Hiesh et al. (2013), the authors extracted statistical features from wavelet transforms (db, level 4) and fed them to the genetic algorithm support vector machine (GA-SVM) on a small dataset. The authors in Agarwal and Singhal (2023) proposed a method using a Fourier-based technique on EEG signals of SB-5 and also used the KW FS method. In Hossein Najafzadeh et al. (2021) and Sahu and Jain (2023), the authors used DWT and MDWT with the (db2) wavelet for preprocessing, focusing on entropy-based features (ShEn, SpEn, and ApEn) and autoregressive coefficients to decompose the EEG signals into distinct rhythms and extracted statistical features with decomposition level 3. The experiments in the aforementioned DWT-based approaches are performed using single or few wavelets. In contrast, in our study, we have used several wavelets with decomposition levels of 5, which is important for capturing the time-frequency features of the signal for the classification of SZ and HC. An advantage of our study is the detailed analysis of multiple wavelets and decomposition levels, allowing us to investigate the role of decomposition level and wavelet selection in SZ detection. However, a limitation of our study is that our analysis was conducted on a single dataset, which may limit the generalizability of our findings. It should be noted that, for clinical purposes, this approach needs to be validated on a larger dataset with more subjects, as well as data from other neurological disorders.

**Table 9:** Performance comparison with the existing approaches.

References	Dataset	Method	Decomposition parameters	FS	Acc
Khare and Bajaj (2021)	81	F-TQWT-based features	SB-6	KW	91.39%
Krishnan et al. (2020)	28	MEMD-based features	IMF-6	RFE	93%
Kumar et al. (2023)	28, 84	SLBP- and HLV-based features	–	Correlation	99.36, 92.85%
Gosala et al. (2023)	84	DWT, CWT, WST-based time, frequency, and time-frequency features	db 1	–	97.98%
Sharma and Acharya (2021)	28	Wavelet-based ↨ 1 norm	level-7	–	99.21%
Hiesh et al. (2013)	10	DWT-based statistical features	db, level-4	GA	88.24%
Siuly et al. (2020)	81	EMD-based statistical features	IMF-7	KW	89.59%
Agarwal and Singhal (2023)	28, 81	Fusion of pattern and statistical	SB-5	KW	99.24, 98.62%
Hossein Najafzadeh et al. (2021)	28	DWT-based AVLSAC, ShEn, SpEn, and ApEn	db2	–	100%
Khare and Bajaj (2022)	84	RVMD-based statistical features	mode-8	KW	92.93%
Sahu and Jain (2023)	28	MDWT-based statistical features	db2, level-3	–	85.71%
Das and Pachori (2021)	28	MIF-based Hjorth parameters	IMF-21	*t*-test	98.9%
Present study	84	DWT-based statistical features by using different wavelets	db 4, coif 1–3, 5 and sym 4	Chi-square	100%

RFE, Recursive feature elimination; KW, Kruskal–Wallis; GA, genetic algorithm; MIF, multivariate iterative filtering; RVMD, robust variational mode decomposition; MDWT, multi-level discrete wavelet transformation.

## 4 Conclusion

In conclusion, our study demonstrates the significance of selecting wavelet decomposition levels and the choice of wavelet type. From our findings, it is clear that higher decomposition levels (specifically four and five) are effective and have achieved a classification accuracy of 100% with the orthogonal wavelets (db 4, coif 1–3, 5, and sym 4). Moreover, our experimental analysis shows that the precision of feature extraction diminishes over longer time windows. In future studies, we plan to explore optimizing the interaction between wavelet decomposition levels and analyze deep learning architectures for the classification of SZ from HC groups. Additionally, we plan to explore the effectiveness of these methods for the diagnosis of other psychological disorders, such as bipolar disorder, depression, and Alzheimer’s disease.

## Data Availability

The original contributions presented in the study are included in the article/supplementary material, further inquiries can be directed to the corresponding author.
